# Automated Oxygen Gradient Ektacytometry: A Novel Biomarker in Sickle Cell Anemia

**DOI:** 10.3389/fphys.2021.636609

**Published:** 2021-03-25

**Authors:** Alina Sadaf, Katie G. Seu, Elizabeth Thaman, Rose Fessler, Diamantis G. Konstantinidis, Holly A. Bonar, Jennifer Korpik, Russell E. Ware, Patrick T. McGann, Charles T. Quinn, Theodosia A. Kalfa

**Affiliations:** ^1^Cancer and Blood Diseases Institute, Cincinnati Children’s Hospital Medical Center, Cincinnati, OH, United States; ^2^Division of Hematology, Cincinnati Children’s Hospital Medical Center, Cincinnati, OH, United States; ^3^Immunopathology Laboratory, Cincinnati Children’s Hospital Medical Center, Cincinnati, OH, United States; ^4^Erythrocyte Diagnostic Laboratory, Cincinnati Children’s Hospital Medical Center, Cincinnati, OH, United States; ^5^Department of Pediatrics, College of Medicine, University of Cincinnati, Cincinnati, OH, United States

**Keywords:** sickle cell anemia, erythrocyte, oxygen gradient ektacytometry, oxygenscan, fetal hemoglobin, F-cell, dense red blood cells, red blood cell

## Abstract

Sickle cell anemia (SCA) is a hereditary hemoglobinopathy with a variable phenotype. There is no single biomarker that adequately predicts disease severity and can be used to monitor treatment response in patients in clinical trials and clinical care. The use of clinical outcomes, such as vaso-occlusive crises (VOC), requires long and expensive studies, sometimes with inconclusive results. To address these limitations, there are several biomarkers under study to improve the ability to predict complications and assess treatment response in both clinical and research settings. Oxygen gradient ektacytometry, also called as oxygenscan, is an assay that measures the effects of deoxygenation and reoxygenation on red blood cell (RBC) deformability and is gaining popularity in SCA research, because it captures the dynamic sickling capacity of a patient’s RBCs as they are subjected to an oxygen gradient under steady shear stress. We describe here the oxygenscan methodology and evaluate the correlation between oxygenscan parameters and more well-known biomarkers of SCA such as fetal hemoglobin (HbF), F-cells, and dense red blood cells (DRBCs). Our data indicate that the oxygenscan curve is affected by all these parameters and the result incorporates the effects of %HbF, %F-cells, RBC hydration, and RBC membrane deformability.

## Introduction

Sickle cell disease (SCD) is an umbrella term for a group of inherited hemoglobinopathies. A single nucleotide mutation in the *HBB* gene, which encodes the β-globin chain of hemoglobin, results in the sickle hemoglobin allele β^S^. Sickle cell anemia (SCA) is used to describe sickle cell disease caused by homozygosity for β^S^ (SS) or compound heterozygosity for β^S^ and a β^0^-thalassemia mutation (Sβ^0^). HbS polymerizes upon deoxygenation causing the red blood cell (RBC) to acquire a “sickle” shape (Steinberg and Rodgers, 2001). Sickle RBCs are markedly less deformable than normal RBCs (Clark et al., 1980), have an increased transit time in the microcirculation of tissues (Vargas and Blackshear, 1982; Du et al., 2015), and demonstrate increased adherence to the vascular endothelium (Mohandas and Evans, 1984, 1985; Papageorgiou et al., 2018). These processes contribute to the major complications of SCD including vaso-occlusive crises (VOC) and chronic organ damage. However, there is wide phenotypic variability in SCD even between patients with the same genotype (Quinn, 2016); therefore, there is a need to develop biomarkers that can reliably identify the risk of complications and assess treatment response (Hoots and Shurin, 2012).

Ektacytometry is a technique for measuring RBC deformability that is used for the diagnosis of RBC cytoskeleton and hydration disorders such as hereditary spherocytosis and xerocytosis (Bessis et al., 1980; Da Costa et al., 2016; Risinger and Kalfa, 2020). In this technique, RBCs suspended in a liquid of known viscosity are subjected to increasing shear stress, or in the case of osmotic gradient ektacytometry (osmoscan), RBCs are suspended in a medium of varying osmolality and subjected to a steady shear stress. RBCs scatter light from a laser beam directed at the suspension, generating a diffraction pattern that can be analyzed to quantify RBC deformability. Under conditions of normal oxygen saturation (normoxia), RBC deformability in SCA is decreased due to the altered cytoskeleton mechanics of irreversibly sickled cells (ISCs), while the osmotic fragility is decreased due to increased surface-to-volume ratio of the dense or dehydrated cells (Clark et al., 1980). Because the percentage of ISCs and dense cells varies between steady state and during VOC, RBC osmoscan also varies in patients over time (Clark et al., 1983; Ballas et al., 1988; Lande et al., 1988; Ballas and Smith, 1992; Lemonne et al., 2013; Parrow et al., 2017). As sickling is caused by deoxygenation, the study of RBC deformability under varying oxygen saturation is promising to be of physiologic relevance. Oxygen gradient ektacytometry, referred to as the oxygenscan, is a next-generation functional assay that measures RBC deformability through an automated cycle of deoxygenation and reoxygenation, demonstrating characteristic features of sickle RBCs (Rab et al., 2019a). As use of the oxygenscan is increasing in sickle cell research, there is a need for standardization of methodology, clinical validation, and correlation with known SCA biomarkers such as fetal hemoglobin (HbF) and dense red blood cells (DRBCs; Rab et al., 2019b, 2020a).

One of the main drivers of polymerization is the intracellular HbS concentration in RBCs (Noguchi et al., 1983). Cellular dehydration mediated by cell membrane cation channels may increase HbS concentration (Brugnara, 2003). Other factors, such as membrane damage due to oxidation (Aslan et al., 2000) and phosphorylation changes (Noomuna et al., 2020), also affect deformability and accelerate RBC lysis. HbF is known to inhibit deoxygenation-induced HbS polymerization (Poillon et al., 1993) and improve RBC deformability likely by improving membrane mechanics and decreasing the number of ISCs (Parrow et al., 2017). However, because HbF is typically unevenly distributed across the population of sickle RBCs, HbF levels do not accurately capture phenotype variability. A fraction of the RBC population with HbF content of 20–25% can survive up to three times longer than RBCs that do not have HbF (Franco et al., 1998). Although low HbF levels have been correlated with increased mortality in SCA (Leikin et al., 1989), the wide variability of F-cells genetically and in response to HbF-inducing medications, may limit the ability of each one of these biomarkers to estimate severity of SCA (Steinberg, 2005; Steinberg et al., 2014; Quinn, 2016).

DRBCs, another biomarker in SCA, are defined as RBCs with density >1.11 g/ml (Fabry and Nagel, 1982) as measured by density-gradient fractionation methods, or percentage of RBCs with a measured mean corpuscular hemoglobin concentration (MCHC) >41 g/dl (Clark et al., 1980). In adults with SCA, %DRBCs have been associated with increased frequency of leg ulcers, priapism, and renal dysfunction, and their decline with hydroxyurea therapy was shown to be independent of the effect of hydroxyurea on HbF levels (Bartolucci et al., 2012).

The oxygenscan curve is a novel laboratory assay that directly measures the sickling propensity of an entire RBC population when challenged with deoxygenation and reoxygenation. Here, we describe its use as a reproducible functional biomarker, demonstrate and discuss the correlation of main oxygenscan parameters with %HbF levels, %F-cells, and %DRBCs, and illustrate results from patients with SCD (all with SS genotype except one).

## Materials and Methods

All procedures were approved by the Institutional Review Board (IRB) at the Cincinnati Children’s Hospital Medical Center and were in accordance with the Declaration of Helsinki. IRB numbers of the studies that provided samples are 2018-0759 and 2018-5,182.

### Oxygen Gradient Ektacytometry

#### General Principles

**Figure 1:**
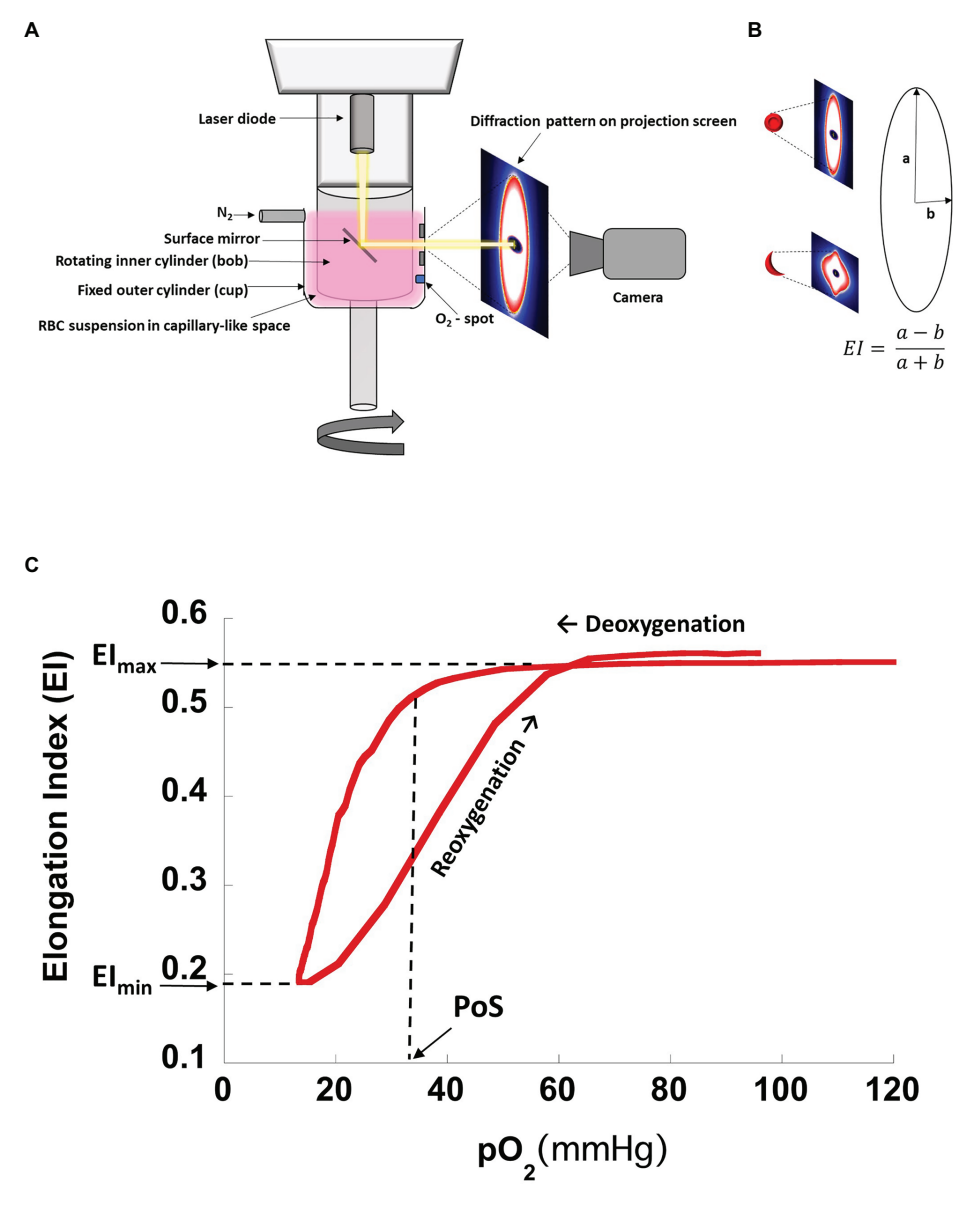
Schematic drawing of oxygenscan setup with representative curve. **(A)** Schematic drawing of oxygenscan. **(B)** Representative diffraction pattern of RBCs in suspension. With deoxygenation and shear stress (30 Pa) in the capillary-like space between the cup and bob, RBCs containing HbS tend to deform to sickled shapes causing the diffraction pattern to change from an elliptical to a rhomboid pattern. Elongation index (EI) is calculated as a ratio of radii along the long and short axis of the diffraction pattern. **(C)** Representative oxygenscan curve of HbS RBCs. Maximum elongation index, EI_max_, is the EI measured at full oxygenation (pO_2_ 100–150 mmHg) and represents baseline RBC deformability in arterial circulation. Minimum elongation index, EI_min_, is the EI measured at the lowest oxygen saturation (pO_2_ < 20 mmHg) and represents RBC deformability in post capillary venules. Point of sickling, PoS, is the pO_2_ at which the EI decreases to 95% of EI_max_ during deoxygenation and represents a patient/disease-status-specific pO_2_ at which HbS polymerization is accelerated and drives the sickling of RBCs that were deformable at normoxia. “Recovery,” i.e., the percentage of EI_max_ reached after reoxygenation (Rab et al., 2019b), may be >100% for certain patients, reaching an EI_max_ value that is higher than the EI_max_ prior to deoxygenation. This is likely related to lysis of less deformable RBCs during the assay, which results in an overall increase in deformability of the RBC population.

The Laser Optical Rotational Red Cell Analyzer (Lorrca®, RR Mechatronics, Zwaag, The Netherlands) oxygenscan measures RBC deformability in terms of the elongation index (EI), which is based on the height and width of an elliptical diffraction pattern (Figures 1A,B). A standardized number of RBCs is suspended in a liquid medium of known viscosity, as detailed below, and exposed to constant shear stress of 30 Pa. The RBC suspension is subjected to one cycle of deoxygenation (1,300 s) through the slow introduction of nitrogen gas followed by rapid reoxygenation (280 s) *via* passive diffusion of ambient air. EI is plotted on a curve against the partial pressure of oxygen (pO_2_; Figure 1C). EI_max_ is the maximum EI measured at full oxygenation (pO_2_ 100–150 mmHg) and represents baseline RBC deformability in arterial circulation. EI_min_ is the minimum EI measured at the lowest oxygen saturation (pO_2_ < 20 mmHg) and represents RBC deformability in post capillary venules. The Point of Sickling (PoS) is the pO_2_ at which the EI_max_ decreases by 5% as deoxygenation proceeds. The PoS represents the patient-specific pO_2_, where HbS polymerization is accelerated and drives the sickling of RBCs that are deformable at normoxia (Rab et al., 2019a). Recovery is calculated as the percentage of EI_max_ reached during reoxygenation and represents the capacity to reverse sickling with reoxygenation.

#### Sample Collection, Storage, and Processing

Peripheral blood samples (at least 1 ml) from patients with SCD were obtained by venipuncture and collected in EDTA tubes. Samples were stored at 4°C overnight, for at least 16 h and at most 32 h, prior to analysis. Twenty-six percent of the samples were processed within 24 h of sample collection. Samples obtained at an outside institution were shipped overnight at 4°C. Care was taken to process samples immediately after removal from 4°C storage.

Prior to analysis, the EDTA tube was gently inverted to allow mixing of plasma with cellular components. RBC count of the sample was determined using the ADVIA® 2120i hematology analyzer (Siemens, Munich, Germany). Next, the volume of whole blood that would yield 200 × 10^6^ RBCs per sample was determined using the following formula.

$$Volumeofsample\;\left(\mu L\right)=200/\left(\begin{array}{l} RBC\;countofsample\\ \;\left(x\;10^{6}\;cells/\mu L\right) \end{array}\right)$$

Sample volume was then added to 5 ml of polyvinylpyrrolidone (PVP) buffer, Iso Oxy (osmolarity 282–286 mOsm/kg, pH 7.35–7.45) with known viscosity of approximately 28 Pa·s. The RBC suspension was gently inverted to allow homogenization. Using a syringe, the sample was drawn up and air bubbles were removed before injection into the machine for analysis. Temperature of the bob (internal cylinder of the Lorrca® oxygenscan) was fixed at 37°C. Camera gain was adjusted to capture the entire diffraction pattern (Figures 1A,B).

### Fetal Hemoglobin

HbF levels were quantified using capillary zone electrophoresis (Sebia Capillarys 2 Flex Piercing System®, Lisses, France). F-cell analysis was performed using an adaptation of a previously described method using multiparametric flow cytometry (Davis and Davis, 2004). Whole blood samples were fixed, permeabilized, and labeled with antibodies to HbF (Invitrogen, Waltham, MA, United States) and CD235a (Glycophorin A, BD Biosciences, San Jose, CA, United States). Flow cytometric analysis was performed using the BD FACSLyric Clinical System (BD Biosciences). Patient samples were accompanied by two separate control samples: a healthy adult and a manufactured high HbF control. F-cell population was quantified as percentage of the total RBCs.

### Dense Red Blood Cells

%DRBCs were measured using an automated analyzer (ADVIA 2120i system), which quantifies %DRBCs by estimating the percentage of RBCs with a measured MCHC >41 g/dl.

### Statistical Analysis

Data from the oxygenscan were exported into R (v i386 4.0.2) to generate curves and determine EI_min_, EI_max_, and PoS. Linear correlations between %HbF, %F-cells, and %DRBCs with oxygenscan parameters were also obtained in R. Oxygenscan profiles were created for individual SCD patients for illustrative results using KaleidaGraph® v 4.1 (Synergy Software).

## Results

**Figure 2:**
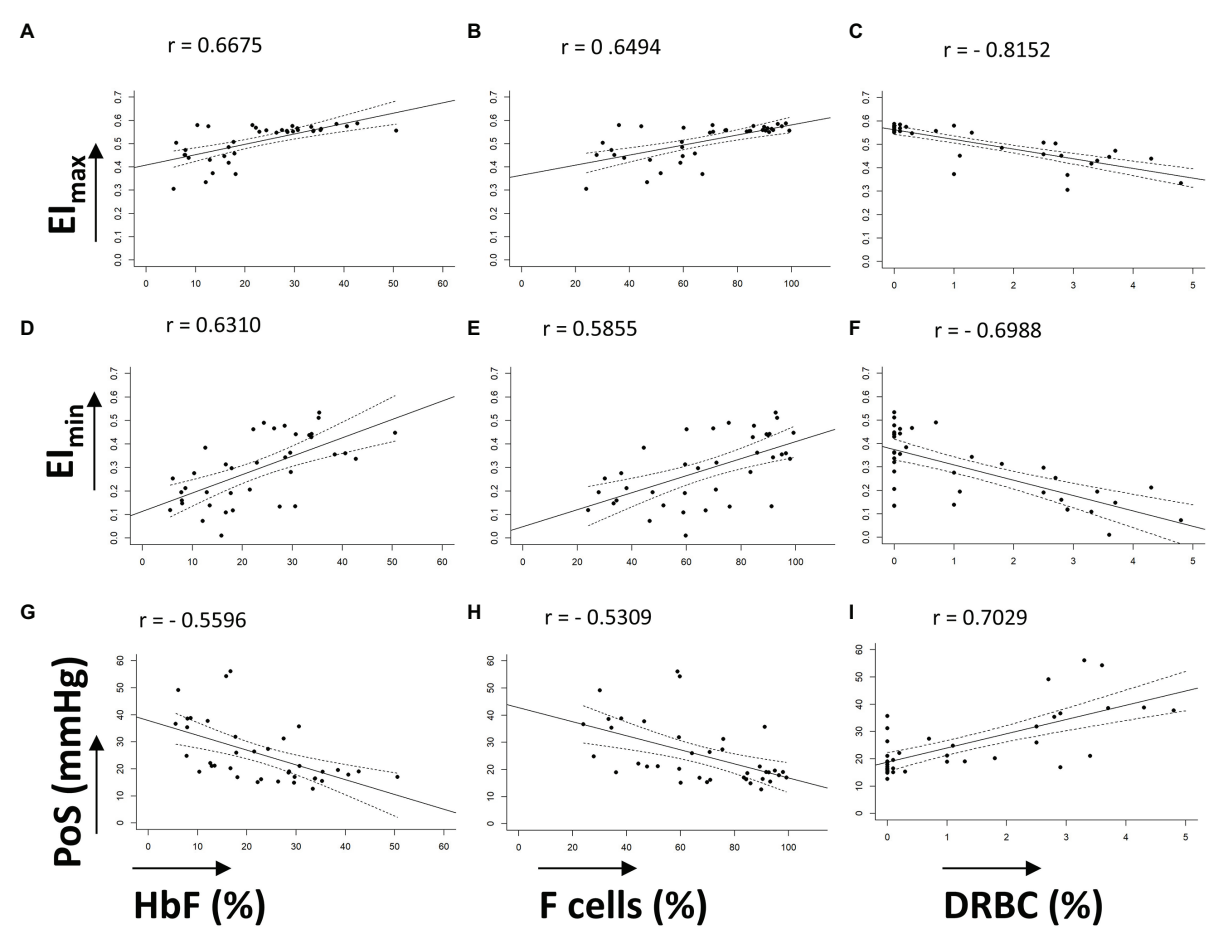
Linear correlations of oxygenscan parameters with known biomarkers. **(A–C)** Linear correlations of EI_max_ with %HbF, %F-cells, and %DRBCs, respectively. **(D–F)** Linear correlations of EI_min_ with %HbF, %F-cells, and %DRBCs, respectively. **(G–I)** Linear correlations of PoS with %HbF, %F-cells, and %DRBCs, respectively. Dashed lines represent 95% CI. All correlations have *p* < 0.001.

Characteristics of the 38 patients with SCD whose samples were tested in this study are summarized in Table 1. All patients were transfusion free for at least 3 months prior to testing. The pediatric cohort of 27 patients, aged from 6 months to 16 years, were not on hydroxyurea. The 11 adult patients were on hydroxyurea therapy with variable compliance. Linear correlation graphs of %HbF, %F-cells, and %DRBCs with EI_max_, EI_min_, and PoS are shown in Figure 2 and results are summarized in Table 2.

**Table 1:** Characteristics of patients evaluated by oxygen-gradient ektacytometry.

	Mean ± SD (range)
Age range (years)	6 months to 48 years
HbSS, *n* (%)	37 (97%)
HbSβ^+^-Thalassemia, *n* (%)	1 (3%)
HbF (%)	23 ± 11.5 [5.6–50.6]
F-cells (%)	67.7 ± 23.1 [24–99.2]
Hb (g/dl)	9 ± 1.2 [6.9–11.4]
RBC (×10^6^/μl)	3.2 ± 0.7 [1.7–4.5]
DRBCs (%)	1.2 ± 1.5 [0–4.8]

**Table 2:** Correlations of oxygenscan parameters with fetal hemoglobin, F-cells, and dense red blood cells.

	EI_min_	EI_max_	PoS
HbF (%)	0.6310 2.17e-05	0.6675 4.70e-06	−0.5596 2.60e-04
F-cells (%)	0.5855 1.13e-04	0.6496 1.03e-05	−0.5309 6.05e-04
DRBC (%)	−0.6988 1.07e-06	−0.8152 4.64e-10	0.7029 8.67e-07

The correlation coefficients (*r*) in black and the values of *p* in bold.

EI_max_ demonstrated a positive linear correlation with %HbF (*r* = 0.6675) and %F-cells (*r* = 0.6494), and a strong negative linear correlation with %DRBCs (*r* = −0.8152; Figures 2A–C). Of note, samples with no DRBC had an EI_max_ that approached 0.6, which is the maximum EI of normal RBCs. When %HbF was more than 20% and %F-cells were more than 70%, the EI_max_ approached the normal maximum value (approximately 0.6), while at lower percentage of F-cells and HbF, a variable decrease of EI_max_ was observed.

EI_min_ showed a positive linear correlation with increasing %HbF levels (*r* = 0.6310) and %F-cells (*r* = 0.5855) and a negative correlation with %DRBCs (*r* = −0.6988; Figures 2D–F). As expected, PoS had a negative correlation with %HbF (*r* = −0.5596), %F-cells (*r* = −0.5309), and a positive correlation with %DRBCs (*r* = 0.7029; Figures 2G–I). Absent DRBCs were not always good predictors for a consistently high EI_min_ or consistently low PoS. It appeared that very high values of %HbF (>30%) and %F-cells (>80%) were required for a reliable decline in the PoS to less than 25 mmHg.

**Figure 3:**
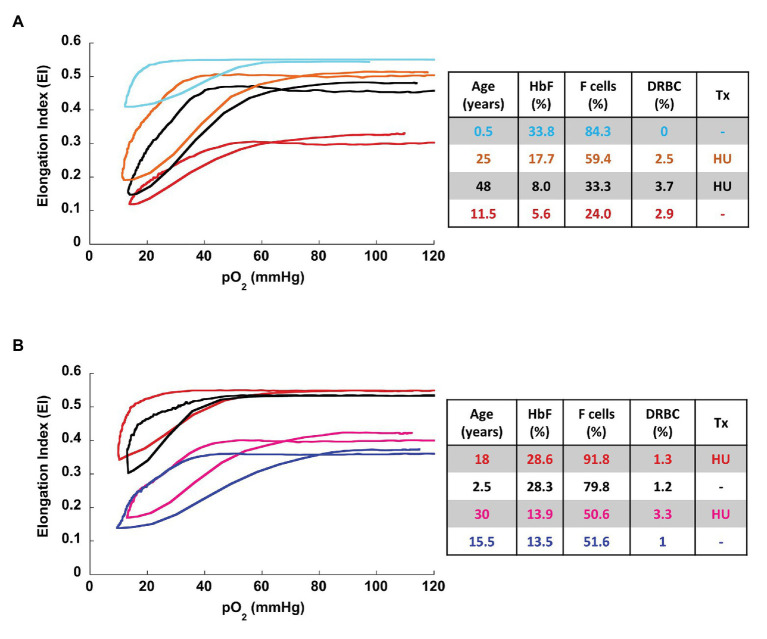
Representative oxygenscan curves. **(A)** Four patients with sickle cell disease with corresponding parameters of %HbF, %F-cells, and %DRBCs. In general, increase fetal hemoglobin content (%HbF and %F-cells) and decrease in %DRBC are associated with increase in EI_max_ and EI_min_ and a decrease in PoS. The oxygenscan profile appears to be affected by the combination of these parameters. **(B)** Two pairs of patients with similar HbF, with and without hydroxyurea therapy. At both high HbF (top pair of curves) and low HbF (bottom pair of curves) hydroxyurea is associated with higher EI_max_ and EI_min_. HU, hydroxyurea; Tx, treatment.

Overall, the oxygenscan profile was influenced by the combination of these classically recognized biomarkers of SCA rather than having a strong association with a single biomarker. Figure 3A illustrates representative oxygen gradient ektacytometry curves from four patients with SCA with varying %HbF, %F-cells, and %DRBCs. A combined increase in %HbF and %F-cells and associated decrease of %DRBCs is necessary to cause an upward shift of the oxygenscan curve resulting in increased RBC deformability, and a lower PoS, indicating an increased capacity to tolerate deoxygenation before sickling is initiated. In contrast, low %HbF and low %F-cells with increased %DRBCs result in lower EI_min_ and EI_max_, indicating a decrease in RBC deformability, as well as a higher PoS, signifying decreased ability to tolerate deoxygenation with sickling initiated at an oxygen pressure that can occur in arterioles in normal physiology.

To illustrate the effect of hydroxyurea on the oxygenscan profile, we identified two pairs of patients with similar fetal hemoglobin values with and without hydroxyurea treatment (Figure 3B). Hydroxyurea appeared to have beneficial effects beyond HbF induction in the deformability parameters of EI_min_ and EI_max_ in both high (28%) and relatively low (13%) HbF values.

## Discussion

Patients with SCA display a wide variation in phenotype, which is inadequately captured by the currently available biomarkers (Quinn, 2016). HbF, one of the best-studied biomarkers in SCA, is used to predict the risk of disease complications and assess response to treatment with hydroxyurea, which is known to induce HbF production in sickle erythropoiesis. However, HbF distribution and induction is widely variable across individuals with SCA, and it is genetically and epigenetically determined (Steinberg et al., 2014). In young patients with SCA, there are distinct patterns of HbF expression and silencing in RBCs (Meier et al., 2011) that confounds the generalizability of HbF and F-cell analysis across age groups. In addition, there has been an increase in treatments that do not rely on HbF induction, such as the Hb-O_2_ affinity modulator voxelotor (Vichinsky et al., 2019), the P-selectin blocker crizanlizumab (Ataga et al., 2017), the amino acid supplement L-glutamine (Anker et al., 2018), as well as cure-targeting strategies such as hematopoietic stem cell transplantation and non-gamma globin-focused gene therapy (Steinberg et al., 2019). The US Food and Drug Administration’s (FDA) approvals for crizanlizumab and L-glutamine were based on a reduction in VOCs without the use of a functional (sickling) assay as an endpoint. Reliance on clinical endpoints such as pain, which are subjective, can lead to long and expensive clinical trials, sometimes with inconclusive results. The phase-3 clinical trial of voxelotor, for example, showed a modest increase in Hb concentration but no effect on the frequency of vaso-occlusive complications (Vichinsky et al., 2019). For these reasons, there has been a call for expanding research into SCD phenotyping and the development of laboratory biomarkers by the U.S. National Heart, Lung, and Blood Institute (Yawn et al., 2014).

Oxygen gradient ektacytometry is a laboratory assay designed to mimic *in vivo* RBC physiology in SCA, i.e., stimulation of HbS polymerization on deoxygenation that decreases RBC deformability, allowing for a direct assessment of sickling (Rab et al., 2019a). This is expected to give oxygenscan an advantage over single parameter assessments, such as HbF or DRBCs, as it integrates the composite effects of multiple physiologic changes in the RBCs, including intracellular HbS concentration, RBC hydration, membrane permeability, and mechanical deformability of the cytoskeleton.

The oxygenscan is a rapid, automated test that uses a small amount (<1 ml) of blood. However, there is a need for intra‐ and inter-laboratory standardization of the methodology as a number of variables may affect the results (Rab et al., 2020a). For example, 2,3-diphosphoglycerate (2,3-DPG) is known to reduce the percentage of oxyhemoglobin S at a given oxygen tension and depletion of 2,3-DPG may reduce sickling (Charache et al., 1970). The intracellular concentration of 2,3-DPG in RBCs is depleted with time (Chanutin and Curnish, 1967), so there is a need to standardize storage time of samples. Although cold storage is important to reduce RBC hemolysis, RBCs stored at 4°C have a minimally increased oxygen affinity after 2 days, which further increases with prolonged storage (Valtis and Kennedy, 1954). Recently, it has been shown that the storage of blood samples at 4°C for greater than 24 h may affect the EI_max_ and EI_min_ in oxygen gradient ektacytometry while the PoS remains fairly stable (Rab et al., 2019a; Boisson et al., 2020). As it was not feasible to process shipped and in-house samples within the same day, we recognize this as a limitation of the current work. To apply uniform conditions, we standardized the analysis time for all samples to 16–32 h from the time of collection (day after the blood draw). We recommend using the same post-collection time for analysis of paired samples when monitoring response to a therapeutic intervention.

During the analysis, the bob is maintained at a physiologic temperature of 37°C. This prevents changes in viscosity caused by variable temperature of the RBC suspension. Oxygen affinity of hemoglobin is also affected by pH with a decrease in oxygen affinity at lower physiologic pH values (Bohr effect; Jensen, 2004). To avoid changes in pH, a standardized PVP buffer, Iso Oxy, is used, which has a physiologic pH of 7.35–7.45. Another important consideration is to use a fixed number of RBCs using the equation described in the methods, because anemia will reduce the viscosity of the RBC suspension. We obtain the RBC count of the sample with an automated flow-cytometry-based hematology analyzer prior to performing the oxygenscan.

The diffraction pattern has an elliptical shape and adjustment of the camera gain is necessary to capture the entire image to determine EI. It should be noted that this approach assumes that as deformability decreases the RBC population acquires a more spherical shape. However, for a population of sickle RBCs, the diffraction pattern on deoxygenation may be rhomboid rather than spherical, so the EI formula may not accurately capture the change in deformability. Further research is needed to better capture the three-dimensional change in diffraction to estimate changes in deformability with greater accuracy.

Rab et al. (2019b) have previously reported on the effects of hydroxyurea treatment and transfusion therapy on oxygenscan parameters. In a mixed cohort of SCD patients with and without hydroxyurea treatment, they demonstrated a significant correlation of oxygenscan parameters with %HbF (EI_max_ *r* = 0.650, *p* = 0.009; EI_min_ *r* = 0.833, *p* < 0.001, PoS *r* = 0.574, *p* = 0.025). Our results on correlation with %HbF are in agreement: EI_max_ *r* = 0.6675, *p* < 0.001; EI_min_ *r* = 0.6310, *p* < 0.001, PoS *r* = 0.5596, *p* < 0.001. The congruence especially for EI_max_ and PoS are striking.

We analyzed data from SCD patients across a wide age range, 6 months to 48 years, and demonstrated correlations of oxygenscan parameters with fetal hemoglobin including %HbF and %F-cells. While %DRBCs have been shown to change independently of HbF in response to hydroxyurea (Bartolucci et al., 2012), we demonstrated a correlation of oxygenscan parameters with both %DRBCs and %HbF. However, although each of the oxygenscan parameters have a linear correlation with each of the known SCD biomarkers examined, the oxygenscan profile is affected by all of them and likely additional ones in a more complex way. For example, patients with similar %HbF with or without hydroxyurea treatment, may have different oxygenscan profiles likely due to changes in RBC hydration caused by hydroxyurea (Figure 3).

We noted that very high values of %HbF (>30%) and %F-cells (>80%) were consistently associated with a lower PoS (<25 mmHg). A highly significant correlation between the %F-cells and the log (%HbF) has been shown before (Marcus et al., 1997). This, in combination with our findings, indicates that a goal of 30%HbF and 80% F-cells may be more appropriate for hydroxyurea therapy or γ-globin targeting gene therapy to have the optimal effect. A recent study in pediatric patients with SCA has demonstrated that an early initiation of hydroxyurea using an individualized, pharmacokinetic-guided dosing strategy can achieve this goal in the clinical setting (McGann et al., 2019). Novel medications that increase Hb-O_2_ affinity may synergize with hydroxyurea to lower PoS (Brown et al., 2020; Rab et al., 2020a). All patients in our analysis had SS genotype, except one patient with Sβ^+^-thalassemia. Further work is required to evaluate the oxygenscan profile of patients with other SCD genotypes, such SC and Sβ^0^-thalassemia, as well as with genetic modifiers such as the hereditary persistence of fetal hemoglobin (HPFH) and α-thalassemia trait. In addition, all patients in this analysis were at their steady state at the time of sample collection. Recently, Rab et al. (2020b) described the correlation of oxygenscan parameters with patients who experienced VOC compared to those who did not and demonstrated that oxygenscan parameters had a predictive value for increased VOC frequency, and oxygenscan curve improvement aligned with response to hydroxyurea treatment. Further research is needed to correlate oxygenscan parameters with the various aspects of SCD phenotype and evaluate changes with new medications being developed and used for SCD management.

## Conclusion

Oxygen-gradient ektacytometry is a reproducible, rapid, and automated functional assay that evaluates the sickling capacity of an erythrocyte population when challenged with deoxygenation and reoxygenation. Although the oxygenscan parameters have an individual relationship with known biomarkers of SCD severity, the oxygenscan profile captures the cumulative effect of those and likely additional factors and may serve as a novel and useful biomarker to evaluate SCD phenotype.

## Data Availability Statement

The raw data supporting the conclusions of this article will be made available by the authors, without undue reservation.

## Ethics Statement

The studies involving human participants were reviewed and approved by Institutional Review Board at Cincinnati Children’s Hospital Medical Center. Written informed consent to participate in this study was provided by the participants or their legal guardians/next of kin.

## Author Contributions

AS and TK performed the data analysis, prepared the figures and tables, and wrote the first draft of the manuscript. KS provided expertise in the performance, standardization, and troubleshooting aspects of the oxygenscan assay. RF, ET, and DK performed the oxygenscan assays. JK performed assays for RBC counts, %DRBCs, and %HbF. HB performed the F-cell assays. RW and PM were principal investigators of trials from which patient samples were obtained. CQ provided expertise in the interpretation of F-cell studies. All authors contributed to the article and approved the submitted version.

### Conflict of Interest

The authors declare that the research was conducted in the absence of any commercial or financial relationships that could be construed as a potential conflict of interest.
